# Draft Genome Sequence of a Nitrate- and Phosphate-Removing *Bacillus* sp., WBUNB009

**DOI:** 10.1128/genomeA.00254-12

**Published:** 2013-02-21

**Authors:** Shreya DebRoy, Pallavi Mukherjee, Sujata Roy, Ashoke Ranjan Thakur, Shaon RayChaudhuri

**Affiliations:** a West Bengal University of Technology, West Bengal, India; b Rajalakshmi Engineering College, Chennai, India; c Techno India University, West Bengal, India

## Abstract

The draft genome sequence (5,868,741 bp) of a nitrate- and phosphate-removing *Bacillus* sp., WBUNB009, isolated from a raw sewage canal in nitrate broth (Himedia M439) with a G+C content of 34.9% is reported. It removes 60.23% nitrate and 96% phosphate within 16 h at 37°C.

## GENOME ANNOUNCEMENT

The contamination of water by nitrates due to anthropogenic activity is a potential hazard throughout the world. Nitrate-induced methemoglobinemia contributes to national infant death rate statistics (1). Application of excess nitrogen-based fertilizers also leads to the percolation of nitrate into subsurface water bodies. Improper disposal of the human and animal waste and the use of unlined drainage and sewerage pipes also add to the nitrate contamination of ground water (2).

Phosphorus is a scarce element with finite reserves on the earth and no synthetic method for its production. Phosphorus is extremely important for plant growth but only a fraction of it is utilized while the rest seeps into water bodies, causing eutrophication. High accumulation of residual phosphate in soil due to excessive use of fertilizers may enhance the downward movement of phosphate, which may eventually reach groundwater (3). Microbial strategies are currently being used for the removal of the excess phosphate load in wastewater because these methods are attractive alternatives to chemical processing (4).

This pure isolate with the ability to remove nitrate and phosphate was isolated from raw sewage canal water in nitrate broth with 2,000 ppm nitrate. This organism reaches a stationary phase at the 6th hour with a generation time of 21 min at 37°C. Its optimum growth is at 30°C and pH 9. The strain is available at the Microbial Culture Collection at the National Centre for Cell Sciences, Pune, India.

This whole-genome sequence was obtained on a 316 chip using an Ion Torrent PGM instrument. The 3,322,254 reads were assembled using the MIRA assembler version 3.4.0. The coverage was 98× with 289 contigs generated. A total of 660 Mb data were sequenced, with 458 Mb data with quality values above 20. All contigs generated by the MIRA assembly were submitted to the RAST (Rapid Annotation using Subsystem Technology) annotation server (5). Mauve-based genome analysis revealed extensive rearrangements compared to those of other reported species of bacilli.

The genes responsible for nitrate metabolism, namely nitrite reductase [NAD(P)H] large subunit and small subunit, nitrate/nitrite transporter, and the respiratory nitrate reductase alpha, beta, gamma, and delta chains, are carried by ANFK01000040. The genes for nitrate/nitrite sensor proteins and the nitroreductase family proteins are carried by ANFK01000058. The genes responsible for phosphate metabolism, like polyphosphate kinase (*ppk*), phosphoesterase, and exopolyphosphatase (*ppx*), are carried by ANFK01000043, and genes for phosphoglycerate transporter protein (*pgtP*) and pyrophosphatase (*ppaX*) are carried by ANFK01000004. The genes for phosphate transport system, consisting of phosphate transport system regulatory protein, phosphate transport ATP-binding protein (*pstB*), and phosphate transport system permease protein (*pstA*, *pstC*), are carried by ANFK01000021. The gene for urease domain protein is carried by ANFK01000002. The strain contains two Walker motifs, which are involved in phosphate transport.

### Nucleotide sequence accession numbers.

This Whole Genome Shotgun project has been deposited at DDBJ/EMBL/GenBank under the accession number ANFK00000000. The version described in this paper is the first version, ANFK01000000.
